# The disproportionate case–fatality ratio of COVID-19 between countries with the highest vaccination rates and the rest of the world

**DOI:** 10.1016/j.ijregi.2023.01.011

**Published:** 2023-01-26

**Authors:** Najmul Haider, Mohammad Nayeem Hasan, Javier Guitian, Rumi A. Khan, David McCoy, Francine Ntoumi, Osman Dar, Rashid Ansumana, Md. Jamal Uddin, Alimuddin Zumla, Richard A. Kock

**Affiliations:** a The Royal Veterinary College, University of London, Hatfield, UK; b School of Life Sciences, Keele University, Keele, UK; c Department of Statistics, Shahjalal University of Science and Technology, Sylhet, Bangladesh; d Division of Pulmonary Critical Care Medicine, Dell Medical School at University of Texas, Austin, Texas, USA; e Institute of Population Health Sciences, Barts and London Medical and Dental School, Queen Mary University of London, London, UK; f Congolese Foundation for Medical Research, Brazzaville, Republic of Congo; g Institute for Tropical Medicine, University of Tübingen, Tübingen, Germany; h Chatham House Centre for Global Health Security, Royal Institute of International Affairs, London, UK; i School of Community Health Science, Njala University, Bo, Sierra Leone; j Department of General Educational and Development, Daffodil International University, Dhaka, Bangladesh; k Division of Infection and Immunity, Centre for Clinical Microbiology, University College London, London, UK; l NIHR-BRC, University College London Hospitals, London, UK

**Keywords:** COVID-19, SARS-CoV-2, Case–fatality ratio, Vaccine equity, Variants of concern, Vaccine roll out

## Abstract

**Objectives:**

The global reported cumulative case–fatality ratios (rCFRs) and excess mortality rates of the 20 countries with the highest coronavirus disease 2019 (COVID-19) vaccination rates, the rest of the world and Sub-Saharan Africa (SSA) were compared before and after the commencement of vaccination programmes.

**Methods:**

A time series model was used to understand the trend of rCFR over time, and a generalized linear mixed model was used to understand the effect of vaccination on rCFR.

**Results:**

By 31 December 2022, an average of 260.3 doses of COVID-19 vaccine per 100 population had been administered in the top 20 vaccinated countries, compared with 152.1 doses in the rest of the world and 51.2 doses in SSA. The mean rCFR of COVID-19 had decreased by 69.0% in the top 20 vaccinated countries, 26.5% in the rest of the world and 7.6% in SSA. Excess mortality had decreased by 48.7% in the top 20 vaccinated countries, compared with 62.5% in the rest of the world and 60.7% in SSA. In a generalized linear mixed model, the reported number of vaccine doses administered (/100 population) (odds ratio 0.64) was associated with a steeper reduction in COVID-19 rCFR.

**Conclusions:**

Vaccine equity and faster roll-out across the world is critically important in reducing COVID-19 transmission and CFR.

## Highlights

• We compared the vaccination rates and case–fatality rates (CFRs) for coronavirus disease 2019 (COVID-19) for various countries on 31 December 2022.
• 260 COVID-19 vaccine doses (/100 population) were administered in the 20 countries with the highest vaccination rates countries are 260.
• In the rest of the world and Sub-Saharan Africa, 152 doses and 51 doses have been administered per 100 population.
• The mean CFR of COVID-19 in the top 20 vaccinated countries decreased by 69%.
• The mean CFR decreased by 27% in the rest of the world and by 8% in Sub-Saharan Africa.

## Introduction

In the early stages of the coronavirus disease 2019 (COVID-19) pandemic, the World Health Organization (WHO) reported a crude case–fatality ratio (CFR) of 3.8% among the first 55,924 laboratory-confirmed cases [1]. Subsequently, systematic reviews reported an estimated CFR of COVID-19 between 2.3% and 3.6% [2], [3], [4], [5]. The global cumulative reported case fatality ratio (rCFR) of COVID-19 increased until the 17^th^ epidemiological week (22–28 April 2020) following the detection of severe acute respiratory syndrome coronavirus-2 (SARS-CoV-2) in Wuhan, China to 7.2%, and then started to decline steadily (2.2% at 31 December 2021) [6].

Vaccination can reduce the CFR of COVID-19. Several vaccines have been approved for emergency use by the US Food and Drug Administration, the European Medicine Agency and the UK Health Security Agency. WHO has also approved the use of a few vaccines across the world. It has been estimated that, 7–28 days after receipt of the second dose of COVID-19 vaccine, infection is reduced by 60–92% [7,8], hospitalizations by 87–94% [7,9] and deaths by 72–100% [7,10] for the Alpha, Beta and Delta variants.

COVID-19 vaccines are not distributed equitably in the world [11]. Although COVID-19 vaccines were developed at an unprecedented rate through the advancement of science and global cooperation, the distribution of COVID-19 vaccines across the world is questionable [12]. Current global vaccination rates are approximately 6.7 million doses per day, and the vaccine distribution is absent or very limited in many low-income countries. Experts anticipated that 80% of the population in low-income countries would not have received any doses of COVID-19 vaccine by the end of 2021 [12], and this has proved to be the case.

As the world faces the third year of the COVID-19 pandemic, as of 31 December 2022, more than 660.35 million confirmed cases of COVID-19, including 6.69 million deaths, have been reported to WHO [13]. Globally, rollout of COVID-19 vaccination has progressed at varying rates, and the impact of mass vaccination on CFR should be explored in order to inform global access to the vaccine. To this end, this study compared the global rCFRs between the 20 countries with the highest vaccination rates (minimum 196.9 doses/100 population), sub-Saharan Africa (SSA) and the rest of the world, before and after commencement of vaccination programmes. In addition, associations between vaccination and other control measures and COVID-19 CFR and excess mortality were explored.

## Methods

### COVID-19 data

The necessary COVID-19-related data, including daily reported new cases, daily reported new deaths, reported total deaths, reported total deaths per million inhabitants and vaccination (number of doses of any COVID-19 vaccine administered/100 population), were collected from the WHO daily COVID-19 situation reports of 210 countries from 1 January 2020 to 31 December 2022 [14]. On 8 December 2020, the first approved COVID-19 vaccine was administered to a human [15]. Twenty-eight days after 8 December 2020 was considered as the cut-off to compare the pre-vaccine period (1 January 2020–5 January 2021) and the post-vaccine period (6 January 2021–31 December 2022). In total, data were obtained from 159 countries for analysis after the exclusion of countries with population <1 million. Of the 159 countries, 44 were from SSA (doses/100 population): Angola (66.6), Benin (31.7), Botswana (118.7), Burkina Faso (22.6), Burundi (0.2), Cameroon (14.3), Central African Republic (42.2), Chad (22.0), Democratic Republic of Congo (17.6), Congo (14.0), Cote d'Ivoire (84.4), Djibouti (30.2), Equatorial Guinea (29.2), Eritrea (0.0), Ethiopia (42.6), Gabon (24.0), Gambia (30.2), Ghana (63.9), Guinea (63.1), Guinea-Bissau (32.0), Kenya (42.7), Lesotho (54.6), Liberia (84.1), Madagascar (8.0), Malawi (31.2), Mali (19.2), Mauritania (85.6), Mauritius (201.1), Mozambique (88.4), Namibia (37.9), Niger (27.4), Nigeria (46.8), Rwanda (189.5), Senegal (15.7), Sierra Leone (71.6), Somalia (48.4), South Africa (63.5), South Sudan (21.2), Sudan (29.3), Tanzania (55.0), Togo (38.4), Uganda (55.7), Zambia (62.9) and Zimbabwe (74.9).

### Identifying the top 20 vaccinated countries

The top 20 countries were those countries that consistently remained in the top 20 in terms of the weekly vaccination rate (/100 population) during the period 5 January 2021–31 December 2022. A count variable was used to identify the top 20 vaccinated countries each week, with a score of 1 if a country was listed in the top 20 countries in a particular week and zero otherwise. This procedure was repeated for each week until 31 August 2022. Finally, the 20 countries with the highest aggregated scores for weekly vaccination rate were selected. The 20 countries with the highest COVID-19 vaccination rates were (doses/100 population): Argentina (247.4), Bahrain (236.2), Belgium (253.8), Cambodia (269.0), Canada (250.4), Chile (319.5), China (243.9), Cuba (381.0), Denmark (223.9), Ireland (220.8), Israel (196.9), Italy (242.9), Japan (300.0), Portugal (270.3), Qatar (282.0), Singapore (261.3), South Korea (250.2), United Arab Emirates (264.0), UK (224.0) and Uruguay (256.4).

### Reported case–fatality ratio

The daily cumulative COVID-19 rCFR was calculated, as described previously [6], as the number of reported COVID-19-attributed deaths per 100 COVID-19 confirmed cases [i.e. rCFR = (weekly reported COVID-19 attributed deaths/weekly reported COVID-19 confirmed cases) x100]. As the numbers of reported cases and deaths both represent a fraction of the total numbers of cases and deaths globally, the term ‘rCFR’ was used [6].

### Excess mortality

Excess mortality is used to describe the number of deaths from all causes during a particular period that is higher than expected under ‘normal’ circumstances [16]. Excess mortality was calculated as the difference between the recorded number of fatalities in a certain week or month (depending on the country) between January 2021 and December 2022, and an estimate of the projected deaths for that time period if the COVID-19 pandemic had not happened [17,18]. There are major difficulties associated with obtaining excess mortality data from many countries; however, ‘Our World in Data’ tracked data from different sources, and the authors were able to extract excess mortality data from 159 countries for the period from 5 January 2021 to 31 December 2022 [19].

### Time series model to predict the trend

Three time series models [i.e. auto-regressive integrated moving average (ARIMA), automatic time-series forecasting model also known as ‘Prophet model’, and simple exponential smoothing (SES)] were used to identify the global trends in COVID-19 rCFR and excess mortality. The details of the SES, ARIMA and Prophet models are discussed elsewhere [6].

### Outcome and predictor variables

Data on selected predictor variables were collected from the World Bank or other United Nations sources and from ‘Our World in Data’, including population density [20], percentage of people aged ≥65 years [21], Gross Domestic Product (GDP) [22], worldwide governance indicators [23], Global Health Security Index [24], and prevalence of obesity [25,26] for analysis. In addition, country-specific prevalence rates of diabetes [26] and cardiovascular disease [26] were included to explain the variation in COVID-19 rCFRs. The ‘Stringency Index’ from the Oxford COVID-19 Government Response Tracker was used [27].

### Empirical evaluation

The ARIMA and Prophet models were assessed by comparing their results with the benchmark model, the SES model [28]. The SES model is the most appropriate non-seasonal model for each series, allowing for any kind of error or trend component. Next, the performances of the time series models were analysed and compared with some commonly used measures to evaluate the significance of predictions, including coefficient of determination (*R^2^*), root mean square error (RMSE) and mean absolute error (MAE).

### Generalized linear mixed model

A generalized linear mixed model (GLMM) with beta distribution was developed to identify whether the explanatory variables were associated with the country's COVID-19 rCFR and excess mortality. The GLMM is an extension of the generalized linear model that allows the analysis of clustered categorical data, as in the case of repeated responses from different subjects [29]. One of the key advantages of the GLMM is that it separates the levels of the models to account for the group effect nesting the lower-level observations. In the present study, there were several observations within the variable ‘locations (country or territory)’. While the location data are assumed to be time-invariant, the independent data are assumed to be universal over the whole study area at a certain time point. The model describes a beta distribution family that has a logit link.

Although the variables were selected carefully, and consistency was maintained with previous publications on the subject [6], the authors were not able to add some potential confounding variables, including the median age of cases in each country. All analyses were undertaken using R Version 3.5.2.2 [30].

### Statistical analysis

**Figure 1:**
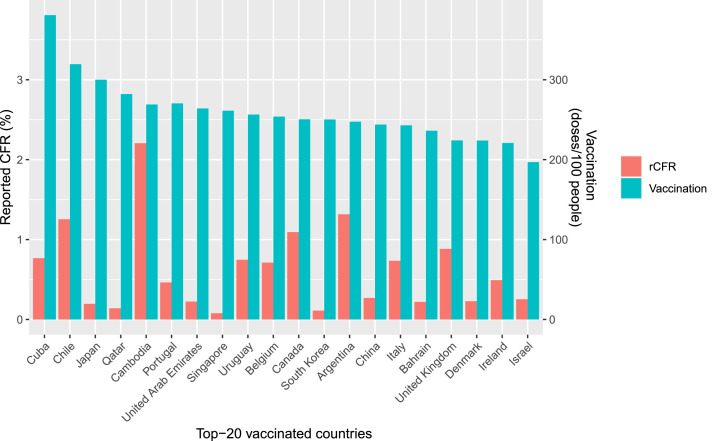
The 20 countries with the highest coronavirus disease 2019 (COVID-19) vaccination rates (reported number of vaccine doses administered/100 population) and the reported case–fatality ratios due to COVID-19. rCFR, reported case–fatality ratio.

**Figure 2:**
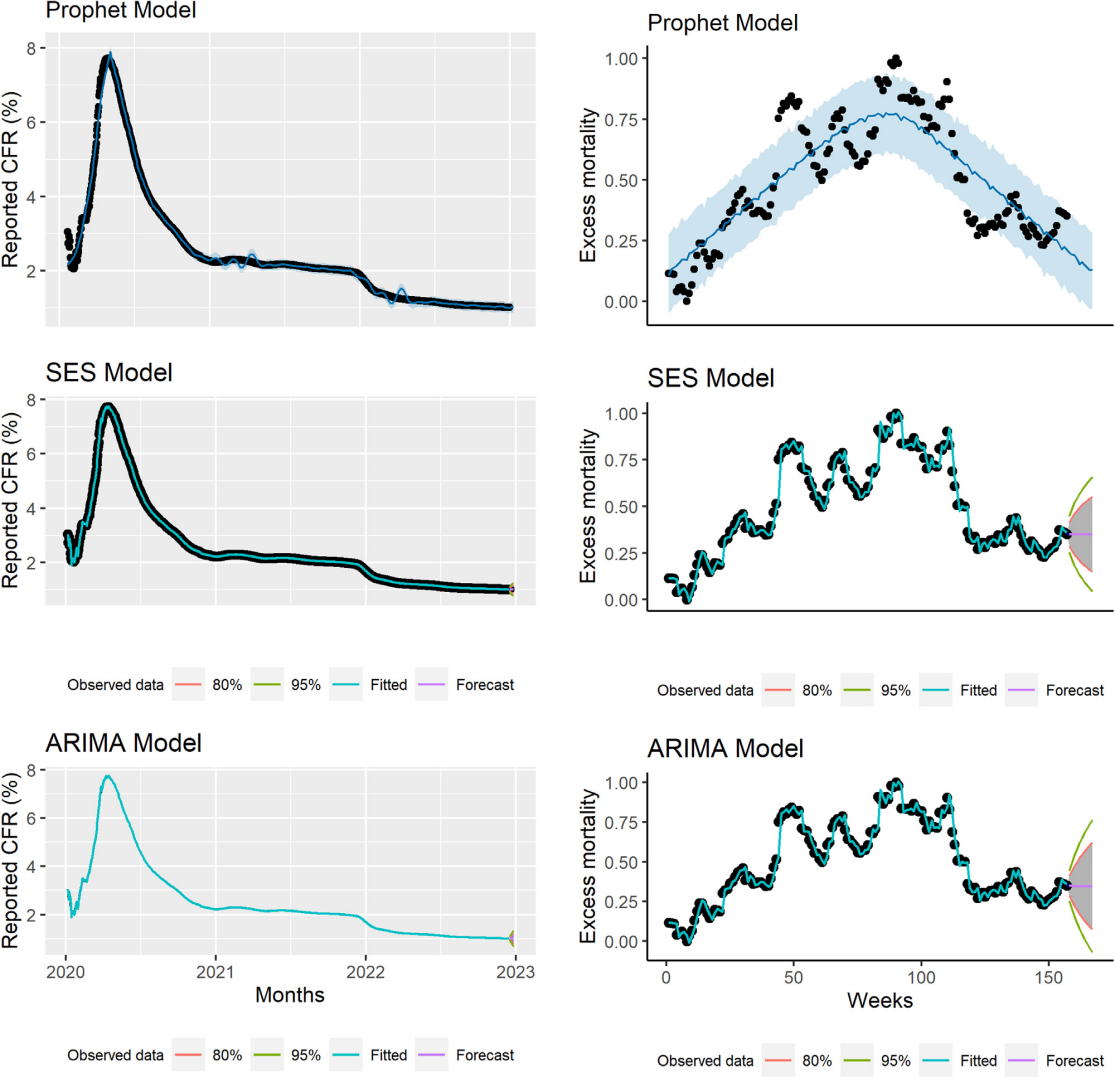
Top: Observed and predicted global daily reported case–fatality ratio (CFR) using the Simple Exponential Smoothing (SES) model. Middle: Observed and predicted daily worldwide daily cumulative reported CFR using the Auto-Regressive Integrated Moving Average (ARIMA) model. Bottom: Observed and predicted daily worldwide daily cumulative reported CFR using the Automatic Forecasting time-series model (Prophet model). Black dots indicate observed data, blue line indicates predicted CFR, and shaded area indicates the 95% confidence interval of predicted CFR.

Summary statistics of vaccine doses/100 population and rCFR were obtained by country for the 20 countries with the highest vaccination rates (Figure 1), SSA and the rest of the world before and after commencement of vaccination programmes, and the mean and standard error (SE) were reported. COVID-19 rCFR and excess mortality changed over time (Figure 2). The use of time series models alone would not enable identification of the reasons for the increasing and decreasing trends in COVID-19 rCFR and excess mortality.

## Results

**Figure 3:**
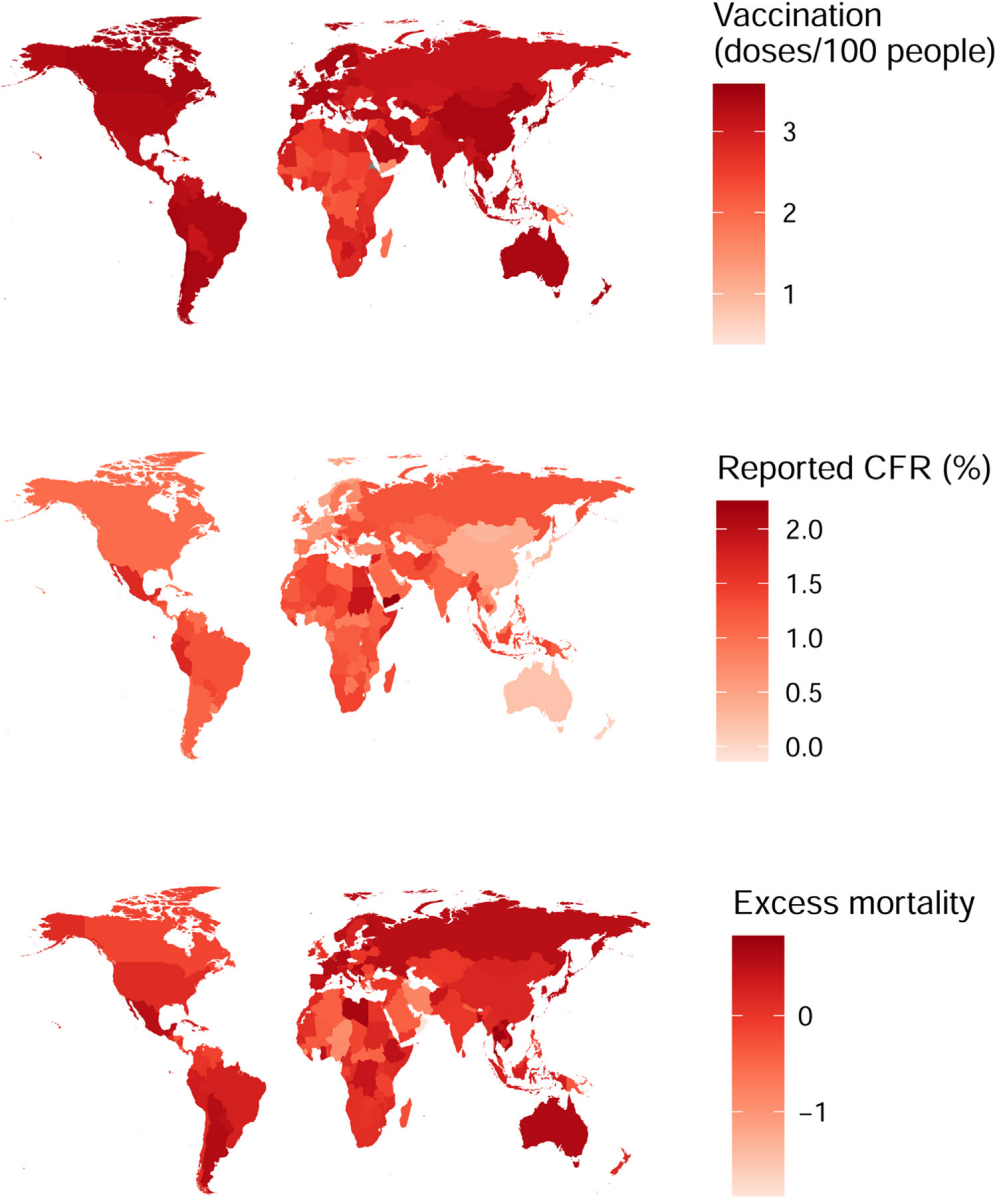
Coronavirus disease 2019 (COVID-19) vaccination rate (reported number of vaccine doses administered/100 population) and the reported case–fatality ratio (CFR) in different countries on 31 December 2022. Darker colour indicates higher vaccination rate or higher case–fatality ratio. An inverse correlation was found between COVID-19 vaccination rate and rCFR (*r*=-0.363, *P*<0.001) and excess mortality (*r*=-0.321, *P*<0.001).

More than 660.35 million cumulative confirmed cases of COVID-19 and 6.69 million COVID-19-related deaths had been documented globally by 31 December 2022, and, on average, more than 165.40 doses of COVID-19 vaccine have been given per 100 population as of 31 December 2022. On average, for every 100 people, 260.33 doses of vaccines have been given in the top 20 COVID-19 vaccination countries. In the rest of the world (including SSA), 152.12 doses of COVID-19 vaccine have been given per 100 population, whereas the number is just 51.21 for SSA (Table 1). rCFR was estimated to be 1.89% in the top 20 vaccinated countries on 5 January 2021, and decreased by 69.31% to 0.58% on 31 December 2022. In the rest of the world, rCFR decreased from 2.34% on 5 January 2021 to 1.72% on 31 December 2022, which represents a reduction of only 26.50% (Table 1). In SSA, rCFR decreased by 7.61% over the same period (1.97 vs 1.82%). Between 5 January 2021 and 30 December 2022, excess mortality decreased by 48.65% in the top 20 vaccinated countries, compared with 62.50% in the rest of the world and 60.71% in SSA (Table 1). The correlation coefficient between vaccination rate (/100 population) and rCFR in different countries on 31 December 2022 is estimated to be -0.363 (*P*<0.001), and -0.321 (*P*<0.001) for excess mortality (Figure 3).

**Table 1:** Reported number of vaccine doses administered/100 population and reported coronavirus disease 2019 (COVID-19) case–fatality ratio (rCFR) in the top 20 vaccinated countries and rest of the world between 5 January 2021 and 31 December 2022 (30 December 2022 for excess mortality).

	Top 20 vaccinated countriesMean ± SE	Rest of the world, including SSAMean ± SE	Global Mean ± SE	SSAMean ± SE
Vaccine doses/100 population (31 December 2022)	260.33 ± 9.35	152.12 ± 6.97	137.67 ± 6.87	51.21 ± 6.26
rCFR by 5 January 2021	1.84 ± 0.39	2.34 ± 0.24	2.27 ± 0.21	1.97 ± 0.19
rCFR by 31 December 2022	0.58 ± 0.12	1.72 ± 0.16	1.59 ± 0.14	1.82 ± 0.20
Decrease in rCFR (%)	69.31	26.50	29.96	7.61
*P*-value (for differences in rCFR between 5 January 2021 and 31 December 2022)	0.003	0.318	0.008	0.309
Excess mortality (deaths/100,000 population) by 4 January 2021	0.37 ± 0.12	0.40 ± 0.05	0.39 ± 0.04	0.28 ± 0.07
Excess mortality (deaths/100,000 population) by 30 December 2022	0.19 ± 0.03	0.15 ± 0.01	0.16 ± 0.01	0.11 ± 0.01
Decrease in excess mortality (%)	48.65	62.50	58.97	60.71
*P*-value (for differences in excess mortality between 4 January 2021 and 30 December 2022)	0.207	<0.001	<0.001	0.022

SSA, Sub-Saharan Africa.

### Factors associated with rCFR and excess mortality

In the GLMM, the estimated effect of each variable is presented as the odds ratio (OR). The reported number of vaccine doses administered (/100 population) [0.64 (0.62–0.65) and 0.96 (0.95–0.98)], GDP [0.79 (0.68–0.91) and 0.92 (0.89–0.96)] and Stringency Index [0.88 (0.87–0.89) and 1.13 [1.11–1.15)] were negatively significantly associated with COVID-19 rCFR and excess mortality, respectively (Table 2).

**Table 2:** Factors associated with reported coronavirus disease 2019 (COVID-19) case–fatality ratio and excess mortality in different counties using a generalized linear mixed model between 1 January 2020 and 31 December 2022.

Variables	Reported case–fatality ratio			Excess mortality		
	Odds ratio	95% confidence interval	*P*-value	Odds ratio	95% confidence interval	*P*-value
Vaccination	0.636	0.620–0.652	<0.001 ***	0.960	0.943–0.977	<0.001 ***
Percentage of people aged ≥65 years	1.189	1.020–1.387	0.027 *	1.164	1.117–1.212	<0.001 ***
Population density	0.878	0.652–1.182	0.390	0.994	0.918–1.076	0.877
COVID-19 tests/1000	0.849	0.829–0.868	<0.001 ***	1.013	1.002–1.023	0.015 **
GDP	0.787	0.682–0.908	<0.001 ***	0.923	0.889–0.959	<0.001 ***
GHSI	1.221	1.043–1.428	0.013 *	0.968	0.928 – 1.010	0.133
WGI	0.986	0.854–1.138	0.848	0.932	0.896–0.969	<0.001 ***
Obesity (%)	1.186	1.054–1.335	0.005 **	1.076	1.042–1.111	<0.001 ***
Stringency Index	0.880	0.867–0.893	<0.001 ***	1.132	1.118–1.145	<0.001 ***
Weeks	1.100	1.044–1.158	<0.001 ***	1.156	1.124–1.187	<0.001 ***
Group name	Variance	Standard deviation		Variance	Standard deviation	
Location (intercept)	0.3103	0.5571		0.01998	0.1414	
Weeks (intercept)	0.0478	0.2186		0.01055	0.1027	
						
Akaike information criterion		-174928.6			-30707.5	
Bayesian Information Criterion		-174826.5			-30605.3	
Root Mean Square Error		0.0001			0.062	
Conditional *R^2^*		0.788			0.513	
Marginal *R^2^*		0.333			0.217	
Intraclass correlation		0.682			0.378	

GDP, Gross Domestic Product; GHSI, Global Health Security Index; WGI, worldwide governance indicators.

Table 2 includes various covariates and the random intercept in the model. The intraclass correlation coefficient of 0.682 was calculated by dividing the variance of the random effect by the total variance. Thus, the spatial unit effects account for approximately 68.2% of the total variance in weekly rCFR, which suggests moderate reliability on location effects on weekly rCFR. It is also to be noted that with the introduction of a random intercept, ‘vaccination’, ‘population density’, ‘GDP’, ‘weeks’ and ‘Stringency Index’ had significant negative effects on weekly rCFR, and ‘percentage of people aged ≥65 years’ had a significant positive effect.

### Trend of global COVID-19 rCFR

**Figure 4:**
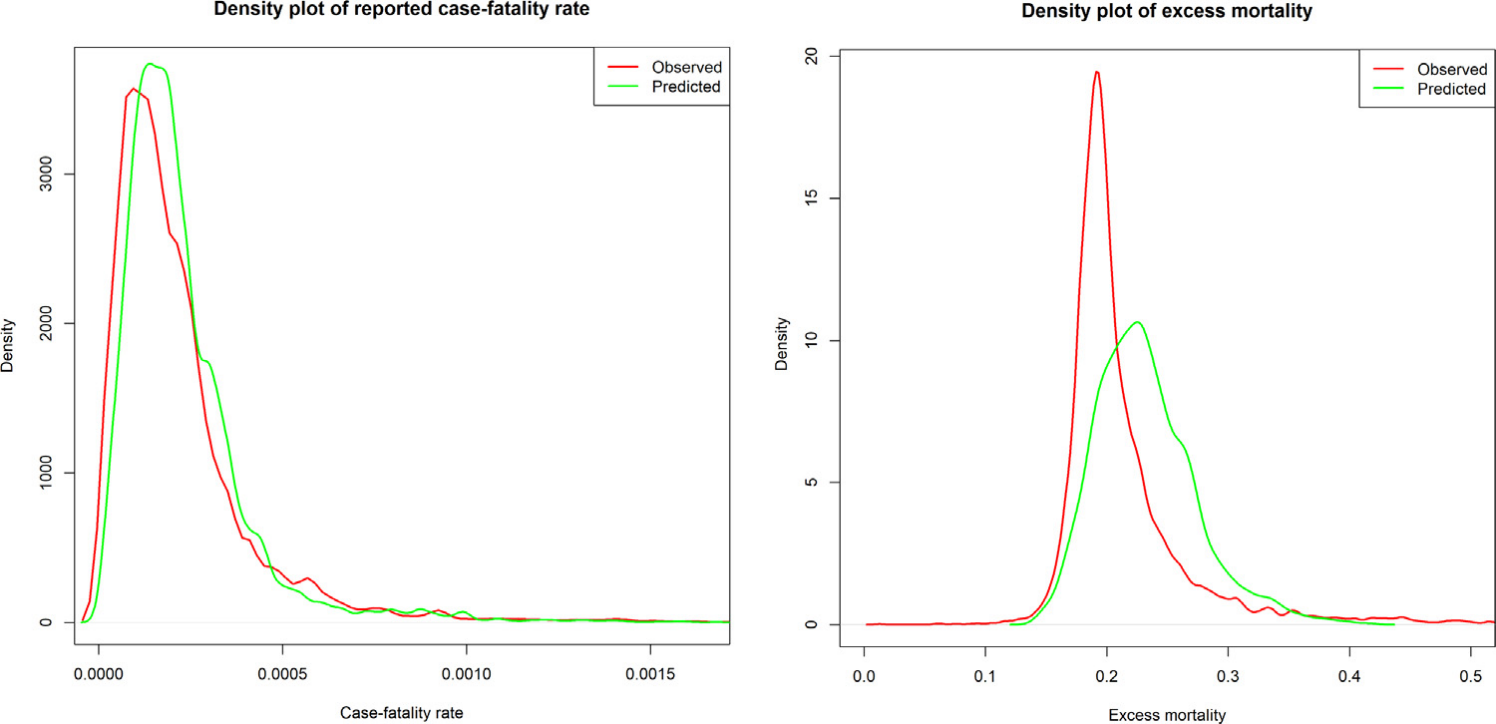
Density plot of observed reported case–fatality ratio (rCFR) and predicted rCFR of for coronavirus disease 2019 (left), and rCFR and excess mortality (right).

The ARIMA and Prophet models found a strong declining trend in COVID-19 rCFR between observed and predicted global COVID-19 rCFR with *R^2^*, RMSE and MAE of 99.97% and 99.50%, 0.029 and 0.113, and 0.008 and 0.059, respectively (Table 3). The observed and predicted rCFR and observed and predicted excess mortality had fair agreement (Figure 4). In terms of accuracy, the ARIMA model performed better that the Prophet and SES models (with better *R^2^*, RMSE and MAE). The coefficient of determination of the ARIMA model was larger and errors were lower compared with the Prophet and SES models. According to the forecast in both models, the ratio of COVID-19 rCFR is expected to decrease considerably in the coming 10 days (Figure 1).

**Table 3:** Summary of the Simple Exponential Smoothing (SES), Auto-Regressive Integrated Moving Average (ARIMA) and Automatic Forecasting Time Series (Prophet) models.

	Reported case–fatality ratio			Excess mortality		
Method and period	*R^2^*	RMSE	MAE	*R^2^*	RMSE	MAE
SES model						
Overall	99.94%	0.038	0.014	96.19%	0.049	0.034
						
ARIMA model						
Overall ARIMA	99.97%	0.029	0.008	96.41%	0.048	0.033
						
Prophet model						
Overall	99.50%	0.113	0.059	75.11%	0.126	0.102

RMSE, root mean square error; MAE, mean absolute error.

The SES, ARIMA and Prophet models used daily cumulative reported case-fatality ratio (rCFR) data.

## Discussion

The global COVID-19 rCFR has been declining since May 2020, stabilizing or increasing slightly with the emergence of different variants of concern (VOCs), particularly the Delta variant [31,32]. Following the rollout of COVID-19 vaccines, rCFR started to decline, although the rates differed between the top 20 vaccination countries (69% decrease) and the rest of the world (27% decrease). In SSA, where the vaccine rollout has not yet reached a satisfactory level, COVID-19 rCFR only decreased by 8%. Excess mortality, on the other hand, decreased by 49% in the top 20 vaccinated countries, 62.5% in the rest of the world and 60.71% in Sub-Saharan Africa. Many factors affect the reduction of rCFR; however, the present results indicate that vaccination and the stringency of control measures influence the reduction in rCFR. A study on the global impact of COVID-19 vaccination showed that vaccination averted 14.4 million reported deaths (or 19.8 million excess deaths) due to COVID-19 in the first year of vaccination (8 December 2020–8 December 2021) [11]. This analysis complements previous findings showing a disproportionate decline in rCFR and excess mortality in countries with various vaccination coverage levels, indicating the importance of equitable distribution of COVID-19 vaccines.

This study found an overall decreasing trend of excess mortality in the postvaccination period. In SSA, in contrast to the negligible decline in rCFR, a marked decrease in excess mortality was noted. Excess mortality during the COVID-19 pandemic was affected by various factors, and not merely by transmission of COVID-19 and vaccination coverage. Some of these factors (e.g. shielding elderly people) may have even contributed to the reduction in excess mortality. The differential impact of these factors made the use of excess mortality an unpredictable estimator of the severity of COVID-19 [33]. This study did not compare excess mortality data at country level. Although differences in data and methodology preclude direct comparisons between the present results and those of the above studies, it should be noted that none of the top 20 countries for excess mortality were in SSA. Although it is beneficial to increase vaccination coverage where possible, this may not be the only reason why the mortality rate in SSA, where the majority of the population are aged <65 years, has decreased markedly since 2021. It is known that survival from COVID-19 is higher in younger age groups [6,34]. The positive association between Stringency Index and excess mortality is surprising and needs further study. An earlier study showed chaotic behaviour of COVID-19 data when compared with the Stringency Index [35], and a weaker relationship with the mortality rate [36]. Excess mortality decreased by 48.6% in the top 20 vaccinated countries, 62.5% in the rest of the world and 60.7% in SSA. Excess mortality showed a different pattern compared with COVID-19 rCFR. This may be because of differences in population age structures between high-income countries and the rest of the world including SSA, rather than the actual impact of lockdown-related control measures estimated in the Stringency Index; there is a need for a well-designed study to investigate this further.

The negative correlation between doses of COVID-19 vaccine given per 100 population, rCFR and excess mortality indicate the benefit of vaccines. Thus, the vaccine has been considered as a pathway out of this pandemic, but strong, innovative policies that ensure fast and equitable distribution are absent [11,12]. Vaccinating the world serves global interests of protecting each other's health and economies [12]. The present analysis showed a large disparity in vaccine rollout between the top 20 vaccinated counties (260 doses/100 population) and low-income countries (e.g. 51 doses/100 population in SSA) by 31 December 2022. In high-income countries, the administration of a third or even fourth COVID-19 vaccine dose is ongoing [37], whereas >60% of the population in SSA have not yet received a single dose (as of 31 December 2022).

COVID-19 vaccination, Stringency Index and GDP were found to be significantly associated with the reduction in COVID-19 rCFR. The main vaccines (mRNA or adenovirus vector) have been found to be highly effective in reducing hospital admissions and deaths, although some vaccines were not very effective at limiting infection [38]. Thus, the vaccine rollout helped high-income countries to reduce the burden of patients in hospitals rapidly, thus limiting the number of COVID-19 deaths. However, in the rest of the world, where vaccine rollout is still far from satisfactory (defined as 70–85% of people are fully vaccinated) [12], CFR has not declined markedly in comparison to the top 20 vaccinated countries. On the other hand, the results from different surveys suggest that natural immunity has reached a state where it is limiting spread and reducing the overall burden of the pandemic in many countries in SSA [39]. More than 28% of people in Brazzaville, Republic of Congo [40], 64.9% of blood donors in Malawi [41] and 74% of community residents in the Central African Republic have been found to have antibodies to SARS-CoV-2 [42]. The country's GDP is another indicator associated with a reduction in COVID-19 rCFR. Countries with higher national income deployed vaccines at a faster rate, which reduced local transmission and the rate of hospitalization, allowing these countries to concentrate on the vulnerable population; these factors acted synergistically to reduce the number of COVID-19-related deaths. Earlier studies have also identified these variables as risk factors for mortality/fatality ratio of COVID-19 [6,43,44].

Equitable distribution of COVID-19 vaccines is crucial to ending the pandemic [11,45]. The circulation of SARS-CoV-2 across the world among the large unvaccinated populations may allow the virus to mutate into a new VOC. Furthermore, many animal species are susceptible to SARS-CoV-2, including mink, primates, rodents, cats and dogs [46]. Several animal species have been linked with the emergence of SARS-CoV-2 VOCs, including dogs for the Alpha variant [47] and rodents for the Omicron variant [48]. Thus, it is important to reduce the circulation of SARS-CoV-2 in human and animal populations in order to avoid further epidemics caused by emergence of new VOCs. Equitable and faster vaccine rollout is key to reducing the circulation of SARS-CoV-2 across the world [11].

The model was adjusted for the number of tests/100 population in each country. Some countries do not report the daily testing number, while some countries share their test numbers irregularly. Testing is an important variable, and failure to adjust for it may result in the identification of spurious relationships. The denominator of rCFR (cases of COVID-19) is entirely predicated on the number of tests that a country has reported. As such, it is possible that the lower rCFRs in higher vaccination countries are simply reflective of greater testing capacity, and thus a larger denominator for the same number of deaths. Thus, only those countries that reported test data regularly were included in the analysis.

This study has several limitations. The main limitation is the dependence of the number of cases on the intensity of testing; as such, countries with low levels of testing missed fatal cases due to COVID-19. In countries where people undergo testing for travelling, working, moving, accessing health care etc., which tend to be higher-income countries where vaccine rollout was faster, large numbers of asymptomatic people are tested daily, which may result in the identification of large numbers of asymptomatic infections. These are then counted as COVID-19 cases, but these people are merely clinically ill. Thus, only some individuals in these countries are actually true cases, because a large proportion of positive cases are asymptomatic and are, therefore, infected. This may increase the denominator of the proportion artificially lowering the CFR. In contrast, it is likely that in SSA, the proportion of apparently healthy individuals who are tested is much lower, leading to apparently lower rCFRs in these countries. The associations reported in this study between COVID-19 rCFR/excess mortality and explanatory variables are statistical associations, and should not be interpreted as causal. Finally, different VOCs of SARS-CoV-2 have different rCFRs, and the VOCs circulated at different times in different countries [32]; it was not possible to adjust for these factors in the analysis.

## Conclusion

More than 260 doses of COVID-19 vaccines have been given per 100 population in the 20 countries with the highest vaccination rates, compared with 152 doses in the rest of the world and 51 doses in SSA as of 31 December 2022. Vaccination is negatively correlated with rCFR (*r*=-0.36) and excess mortality (*r*=-0.32), which is likely to reflect a contribution of vaccines to the reduction in COVID-19-related deaths. Excess mortality and COVID-19 rCFR have continued to decline since February 2021, although at a disproportionate rate between the top 20 vaccinated countries and the rest of the world. However, rCFR has decreased dramatically (69.3%) in the 20 countries with the highest vaccination rates (70%) and quite markedly in the rest of the world (30.0%), whereas it has only decreased by 7.6% in SSA. COVID-19 vaccination, Stringency Index and GDP were associated with a reduction in COVID-19 rCFR. Vaccine equity and faster rollout across the world is critically important to reduce COVID-19 transmission and CFR.
